# Descendant root volume varies as a function of root type: estimation of root biomass lost during uprooting in *Pinus pinaster*

**DOI:** 10.3389/fpls.2013.00402

**Published:** 2013-10-23

**Authors:** Frédéric Danjon, Joshua S. Caplan, Mathieu Fortin, Céline Meredieu

**Affiliations:** ^1^INRA, UMR1202 BIOGECOF-33610 Cestas, France; ^2^Université de Bordeaux, UMR1202 BIOGECOF-33610 Cestas, France; ^3^Bryn Mawr College, Bryn MawrPA, USA; ^4^AgroParisTech, UMR1092 LerfobNancy, France

**Keywords:** root system architecture, forest trees, 3D digitizing, *Pinus pinaster*, uprooting, structural root biomass, fractal branching analysis, biomechanics

## Abstract

Root systems of woody plants generally display a strong relationship between the cross-sectional area or cross-sectional diameter (CSD) of a root and the dry weight of biomass (DW_d_) or root volume (V_d_) that has grown (i.e., is descendent) from a point. Specification of this relationship allows one to quantify root architectural patterns and estimate the amount of material lost when root systems are extracted from the soil. However, specifications of this relationship generally do not account for the fact that root systems are comprised of multiple types of roots. We assessed whether the relationship between CSD and V_d_ varies as a function of root type. Additionally, we sought to identify a more accurate and time-efficient method for estimating missing root volume than is currently available. We used a database that described the 3D root architecture of *Pinus pinaster* root systems (5, 12, or 19 years) from a stand in southwest France. We determined the relationship between CSD and V_d_ for 10,000 root segments from intact root branches. Models were specified that did and did not account for root type. The relationships were then applied to the diameters of 11,000 broken root ends to estimate the volume of missing roots. CSD was nearly linearly related to the square root of V_d_, but the slope of the curve varied greatly as a function of root type. Sinkers and deep roots tapered rapidly, as they were limited by available soil depth. Distal shallow roots tapered gradually, as they were less limited spatially. We estimated that younger trees lost an average of 17% of root volume when excavated, while older trees lost 4%. Missing volumes were smallest in the central parts of root systems and largest in distal shallow roots. The slopes of the curves for each root type are synthetic parameters that account for differentiation due to genetics, soil properties, or mechanical stimuli. Accounting for this differentiation is critical to estimating root loss accurately.

## INTRODUCTION

Root system architecture is one of the primary aspects of plant structure insofar as it influences plant anchorage in the soil and the way plants absorb water and nutrients (Lynch, 2005). However, far less is known about root system architecture than about aboveground architecture because roots are almost entirely hidden in the soil (Böhm, 1979). Due to their spatio-temporal distribution, coarse and fine roots are not studied in the same way, with the categories generally divided at 2 mm diameter (Böhm, 1979). Coarse root distribution varies greatly as a function of position in the root system, whereas fine roots have a more homogeneous distribution at the stand level. Improved methods for quantifying coarse root system architecture (CRSA) of entire trees would therefore be valuable for a number of applications, ranging from studies of tree biomechanics to those of root carbon sequestration (Brunner and Godbold, 2007).

Although there are techniques of growing plants that allow for relatively easy access to roots, these methods often alter root structure and function (Poorter et al., 2012). For example, CRSA is largely altered by growth in a container as soon as the roots reach the wall of the pot. Moreover, soils are complex media and it is nearly impossible to accurately reproduce soil structure in pots. While measurements made on containerized roots may be useful in some contexts, the role of roots in other contexts, such as studies of ecosystem functioning, can only be studied in natural environments (Danjon et al., 2013). Similarly, transparent interfaces placed in the soil give only partial information on CRSA and can modify root growth. Although larger pots decrease the problem of roots intersecting container walls and allow roots to be scanned with computed tomography (CT; Mooney et al., 2012), root system size is still quite limited and only partial information can be inferred with respect to the root system architecture of mature trees. In the field, ground-penetrating radar (GPR) shows promise as a non-invasive measurement technique, but, to date, it has only been used for stand level biomass estimations (Butnor et al., 2003) and in methodological studies (Danjon and Reubens, 2008). Therefore, the root architecture of larger plants should ideally be studied in the field using excavated or uprooted root systems (Danjon and Reubens, 2008).

However, coarse root field studies are time-consuming and data are not available for complete root systems because roots break while being removed from the soil. In non-cultivated soils, roots have to be disentangled from the roots of other plants, stones, or woody debris. Excavating soil from roots causes fewer roots to break than pulling roots from the soil, e.g., with heavy machinery. Retrieving broken roots after mechanical uprooting is time-consuming and not always successful. However, excavation is often prohibitively time-consuming when manual tools are used (Puhe, 1994). Excavation is more rapid when done with pressurized water (e.g., Richardson and Dohna, 2003; Tarroux et al., 2010), but requires special equipment and conditions, such as a water source, sloping ground, and shallow rooting. Roots growing under large neighboring trees cannot be recovered. Lost roots result in an under-evaluation of biomass at the stand level, which might be substantial. Perhaps the largest impact on analysis is when individual root system architecture is studied. For example, in a study of susceptibility to windthrow (Danjon et al., 2005), the relative volume of sinkers leeward of the tree was dramatically underestimated if a large sinker was lost in that sector. Resources for removal are always limited, resulting in a trade-off between the proportion of the root system that can be recovered and the number of root systems that can be accessed.

For a given species, a strong relationship between cross-sectional area (CSA) or cross-sectional diameter (CSD) and descendant root biomass (DW_d_) or volume (V_d_) is assumed in numerous papers (Nielsen and Hansen, 2006). Based on this, techniques of fractal branching analysis (FBA) have been developed to assess CRSA features. Generally, architectural parameters are measured on a small sample of excavated branches, and related to the proximal CSA of all second-order roots (van Noordwijk et al., 1994). There are related techniques for estimating root biomass lost during excavation (e.g., Heth and Donald, 1978).

Fractal branching analysis is based on the assumption that a root system is comprised of self-similar substructures that have consistent tapering and branching properties (van Noordwijk et al., 1994). FBA has been largely used to model the structure of root branches from their proximal diameters. FBA is based on a low number of parameters, including root taper, the CSA shared between the main root and its branches, and inter-lateral branching length. The relationships established in FBA literature demonstrate that there is a basis for estimating missing root volume from CSDs.

Present methods for estimating root biomass lost during excavation are based on the relationship between the CSD and the dry weight (DW_d_) of all descending roots for a fairly intact branch (Whittaker and Woodwell, 1968; Heth and Donald, 1978; Le Goff and Ottorini, 2001). Once known, this relationship can be applied to diameters at broken ends of roots to estimate the missing biomass.

Root systems are generally composed of a set of distinct root forms, which researchers have quantitatively differentiated into root types (woody plants are reviewed by Danjon et al., 2013). To study phosphorus uptake in a crop plant (*Phaseolus*), Rubio and Lynch (2007) defined five root categories: the taproot, lateral roots branching from the taproot, basal roots, laterals originating from basal roots, and roots originating from the hypocotyl. Plants can also grow roots from shoots (Zobel and Waisel, 2010). At least four types of roots were used by Köstler et al. (1968) to describe root systems in temperate forest trees: the taproot, shallow roots, secondary sinkers, and oblique roots. Collet et al. (2006) defined five root types in *Quercus petraea* seedlings. Jourdan and Rey (1997) classified roots of *Elaeis guineensis* using seven categories. These were based on branching orders, but included subclasses for orientation (horizontal or vertical) and depth (shallow or deep). Finally, Danjon et al. (2005) used nine root categories to assess relationships between root architecture and wind-firmness in mature *P. pinaster* root systems (see below). A subset of six of these root categories was used to perform an in depth phenotyping of RSA in *Robinia pseudoacacia* seedlings (Khuder, 2007).

Fractal branching analysis models have tended to treat all roots as a single type, which may be one reason why they have had poor predictive ability (Van Noordwijk and Purnomosidhi, 1995; Danjon and Reubens, 2008). One exception in the FBA literature is Richardson and Dohna (2003); although they did not assess root type explicitly, they showed that FBA parameters do vary as a function of root diameter. Kalliokoski et al. (2010) showed that root tapering could be larger in shallow roots (especially in the zone of rapid taper, ZRT) than in oblique roots, and could decrease with branching order. Nielsen and Hansen (2006) also fitted separate equations between proximal CSA and DW_d_ for horizontal and vertical roots.

Most studies have pooled all roots to estimate missing biomass or volume. However, cases in which DW_d_ or V_d_ were estimated when stratified by root category found stronger relationships. These includes cases in which CRSA has been incorporated in an approximate way, such as an analysis using three diameter classes (Le Goff and Ottorini, 2001). Given that branching and tapering parameters may vary as a function of root type (Danjon and Reubens, 2008), estimates of missing volume or biomass may be more accurate if they take such information into account. It should be noted that the relationship between CSA and DW_d_ is strong partially because root tissue density tends to vary little among root types (Danjon et al., 2006). However, the relationship can have a large inter-stand and inter-species variability (Nielsen and Hansen, 2006; Kalliokoski et al., 2010).

The objectives of this study were (1) to test the hypothesis that the root volume originating from a section varies as a function of root type and (2) to present and apply a new method of assessing the root volume or biomass lost during uprooting. This method avoids the process of collecting and weighing roots, other than to determine root wood density, if required. We used a database of 3D root system architecture that was compiled from a stand of *P. pinaster* trees over time (trees were 5, 12, or 19 years when uprooted). The database included 49 trees, 11,000 roots, and 60,000 root segments. Relationships between CSD and descendant volume were derived for all intact roots in each of 10 architectural types. These relationships were then applied to the broken tips of all root axes to estimate missing volumes.

## MATERIALS AND METHODS

### ROOT SYSTEM DATASETS

The root systems used in this study came from the control plot in a fertilization × irrigation experiment; the design is described thoroughly in Trichet et al. (2008). The experiment took place on a 5.6 ha stand of *P. pinaster* in Pierroton, France, 20 km south-west of Bordeaux. In that region, mean annual rainfall is 850 mm and mean annual temperature is 13^°^C. The water table generally fluctuates close to the soil surface during rainy winters, but sinks to 1.5 m depth in late summer. The experimental plot was 60 m asl and was underlain by a moderately humid, sandy spodosol, with a discontinuous deep hard pan at approximately 70 cm depth. The plot was surrounded by 0.5–1.2 m deep ditches.

In spring 1993, a field was prepared by first removing stumps that remained from a clear-cut, and then plowing the soil to 0.3 m depth. One-year-old *P. pinaster* seedlings were subsequently planted at 2 m × 4 m spacing. Seedlings were of local provenance and were in 200 cm^3^ turf plugs before planting. Major storms damaged the stand in December 1999 and February 2009. The first storm toppled 20% of trees in the control plot; these were later straightened and secured with cables for 2 years. Trees were harvested for root architectural analysis when trees were 5, 12, and 19 years old (**Table 1**). The respective datasets will be referred to as L5, L12, and L19.

**Table 1:** Characteristics of the three root architecture datasets used for regressions and estimation of missing root volume.

Variable		Unit			
Dataset name			L5	L12	L19
Tree age		Year	5	12	19
Total trees			30	12	7
DBH		cm	5.8	17.4	28.5
Standard deviation DBH		cm	1.2	3	2.6
Basal diameter threshold		cm	0.2	0.2	0.5
Depth limit definition	Shallow roots	cm or %	-15	33%	33%
	Deep roots	cm or %	-40	60%	60%
Mean root system	Length	cm	3988	11915	29517
	Volume	cm^3^	2731	33527	136977
Total	No. roots included		2877	3851	4009
	No. segments included		22740	18176	21923
Selected for analysis	Segments (QR_0_)	%	15.7	16	26
	Axes (QR_0_)	%	17.4	19.2	37
	Segments (LR_1_)	%	14.6	16.3	18.5
	Axes (LR_1_)	%	17.3	19	23.3

*Depth limits were used to classify each segment by root type; fixed depths were used for L5, whereas depth limits corresponding to a percentage of maximal rooting depth by tree were used for L12 and L19. Ages were based on the dates that plants germinated.*

In the L5 dataset, trees were selected from across the diameter at breast height (DBH) range represented in the stand. Trees were uprooted by pulling the stem upward with a logging crane after loosening the soil with hand tools. Further details of measurement and uprooting are given in Danjon et al. (1999a),b. In the L12 dataset, the sample consisted of seven of the largest DBH trees, as well as five trees that spanned the range of DBH values in the stand (Danjon et al., 2007). In L19, only the largest, most dominant trees were sampled (Augusto et al., 2013). In L12 and L19, trees were uprooted after removing soil from shallow roots with an air-lance, loosening the soil between the shallow roots with the bucket of a mechanical shovel, and then pulling the stem vertically. Details of uprooting and measurement for the L12 and L19 datasets are given in Danjon and Reubens (2008).

Digitizing in 3D was performed using a Fastrack positional measurement system with a Long Ranger magnetic source (Polhemus, Colchester, VT, USA). Each root was divided into approximately 20 cm long segments so as to record changes in direction or diameter, as well as branching points. Spatial coordinates (*X*, *Y*, and *Z* values) were measured at the base of each root axis and at the end of each segment with the digitizer’s stylus. The largest and smallest root diameters were also entered for each of these cross sections. In recording L5 and L12, diameters were measured with an analog caliper (0.5 mm resolution). In L19, diameters were measured with a Mitutoyo 700-126 or 700-128 plastic digital caliper (0.1 mm resolution). All excavated roots with basal diameters larger than a given threshold were digitized (**Table 1**). The taproot was considered the first-order root.

Several additional features were recorded during measurement. These included the positions of intra-tree root grafts (except for L5), ball-shaped growths of unknown origin that occasionally appeared on roots, and forks, which we defined as multiple, higher-order roots extending from a single, lower-order root. We distinguished normal forks from traumatic forks, as multiple root axes often form where tips are killed or roots cut (note that these are called traumatic reiterations elsewhere; Collet et al., 2006; Danjon et al., 2013).

### ANALYSIS OF ROOT ARCHITECTURE

**FIGURE 1:**
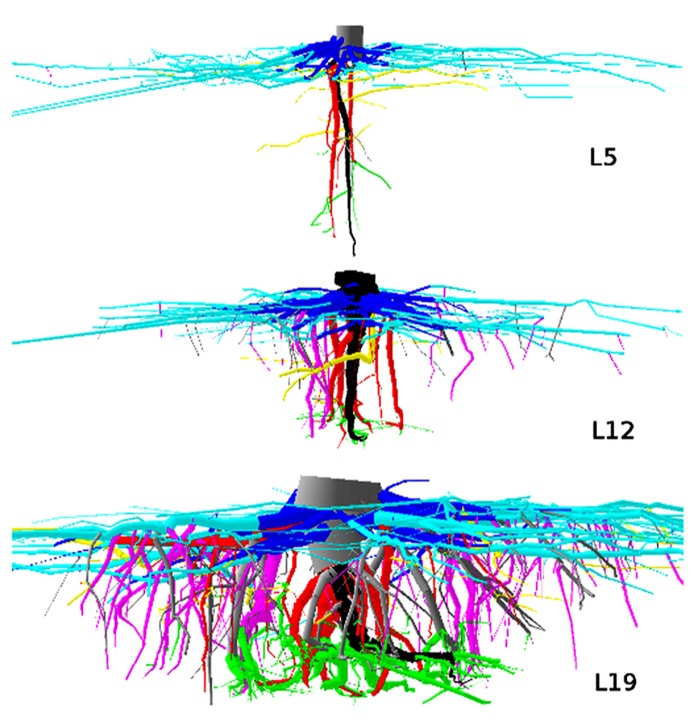
**3D reconstruction of one tree from each dataset, side view**. L5: 2.3 m image width, tree 16, 10.7% root volume lost. L12: 3 m image width, tree 725, 6.9% root volume lost. L19: 3 m image width, tree 4601, 3.51% volume root loss. View is from the West. Segments are coloured as a function of their root type: dark gray, root stump; black, taproot; dark blue, shallow roots in the zone of rapid taper (ZRT); light blue, shallow roots beyond ZRT; red, sinkers from ZRT; magenta, sinkers beyond ZRT; yellow, intermediate depth horizontal roots; green, deep roots; gray, oblique roots above the deep limit. Shallow roots are clipped.

**FIGURE 2:**
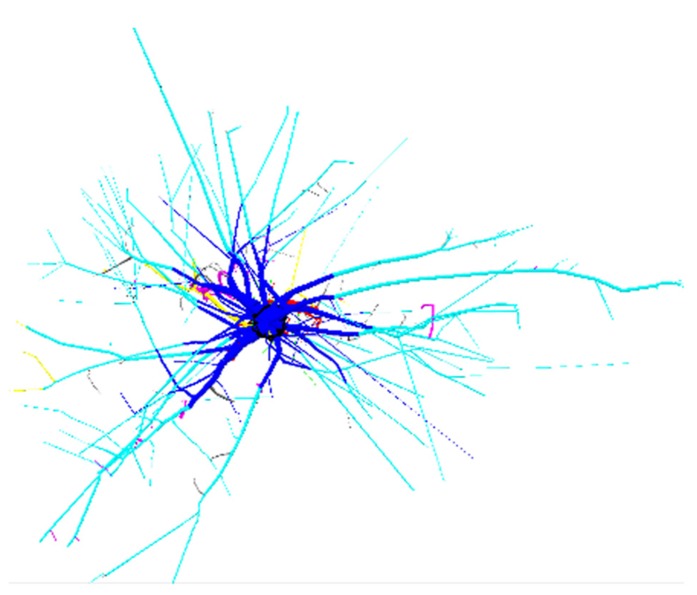
**3D reconstruction of one tree from dataset L12, top view**. 4 m image width, tree 725. Coloration follows **Figure 1**. Shallow roots are not clipped.

We performed a quantitative architectural analysis (Barthélémy and Caraglio, 2007) to determine the functional role of individual roots with respect to maintaining the stability of the tree. This entailed matching segments to one of nine structural classes that occur in tree root systems; the classes are based largely on position and orientation, and therefore correspond to the biomechanical properties that they convey (Danjon et al., 2005; Danjon and Reubens, 2008). An additional class for the higher-order shallow roots was added. Classes were defined as follows (**Figures 1** and **2**):

(1) Root stump; the portion of the taproot that has a large diameter, and where most shallow lateral roots originate (Nicoll et al., 1995).
(2) Taproot; the largest root originating at the distal part of the root stump and growing in a vertical direction.
(3) Shallow roots in the ZRT; the proximal part of second-order shallow roots (also third-order roots if they originate from a fork).
(4) Shallow roots beyond the ZRT; the distal part of second-order shallow roots (also third-order roots if they originate from a fork).
(5) Sinker roots extending from the first-order root or from the ZRT of shallow roots.
(6) Sinker roots extending from shallow roots beyond the ZRT.
(7) Intermediate-depth horizontal roots.
(8) Deep roots; those that originate below a threshold value.
(9) Oblique roots that originate above the deep-root limit.
(10) Shallow roots with branching order >2 (or >3 in the case of forks).

The limit between horizontal, oblique and vertical roots was set to 30^°^ and 60^°^, respectively. The depth limits between shallow, intermediate-depth and deep roots are indicated in **Table 1**. We used fixed limits for L5 and a percentage of maximal depth for L12 and L19, according to Danjon et al. (2005). In L5, the limit for the ZRT was fixed to a radial distance of 2.5 times the DBH. In L12 and L19, the ZRT extended from the root base to the last segment for which the taper from root origin was larger than 2% per cm for L12 and 1.25% per cm for L19 (Danjon et al., 2005).

Using AMAPmod (Pradal et al., 2008), we computed several characteristics for each of the 60,000 distal cross sections in the database or the roots originating from them:

(1) Cross-sectional diameter measured over bark; where cross sections were elliptical and two diameters were measured, CSD was computed as the quadratic mean.
(2) Total root volume originating from the section, V_d0_.
(3) Root volume originating from the section, but including only segments with diameters larger than 1 cm, V_d1_.
(4) Sum of the distal cross-sectional areas (cross sectional areas; break points) of all root segments descendant from the section, ΣCSA_end_.
(5) Sum of descendant root graft surfaces (Danjon et al., 2005), ΣS_graft_.
(6) Sum of the distal CSAs of roots ending in a traumatic fork, ΣCSA_fork_.

We used these computations to determine the relationship between the CSD or the CSA and the total root volume originating from that cross-section, excluding segments with non-characteristic taper:

(1) Bases of large roots are often thickened for reinforcement; we excluded the most proximal segment of each root in L12 and L19.
(2) *Pinus pinaster* roots often form intra-root-system grafts, where a large root can taper abruptly after crossing a small root, and the small root increases after the graft (Danjon et al., 2005). Therefore, segments for which ΣS_graft_ > CSA/20 were excluded.
(3) When a root is cut or dies at a given point and a traumatic fork is formed, some root volume is lost and the architecture is disturbed. Therefore segments for which ΣS_fork_ > CSA/20 were excluded.
(4) Segments with oversized diameters that corresponded to growths were excluded.

The L5 selection of segments for model training did not include sinkers beyond the ZRT, and included very few deep root segments, at this age, these roots were fine and were not measured.

### STATISTICAL ANALYSIS

We assessed the relationship between CSD and the two measures of V_d_. First, we assessed structural roots defined as sections larger than 1 cm, segments in the root stump, and segments bearing branches without break points >1 cm in diameter:

$$\sqrt{V_{{d1}_{ij}}}=\lambda_{i}+\gamma_{i}{CSD}_{ij}+\varepsilon_{ij}\,\;\;({LR}_{1})$$

where V_d1ij_ is the volume of section *j* in root type *i*, λ_i_ is the effect of root type *i*, γ_i_ is a root type-dependent slope, and ε_ij_ is a residual term. A simplified LR_1_ model was also generated, in which four root types were specified rather than ten. In some studies, log–log transformations have been used to obtain a linear relationship between CSD or CSA and DW_d_ (Le Goff and Ottorini, 2001; Nielsen and Hansen, 2006). For our database, this transformation did not yield Gaussian-distributed residuals, whereas the square root transformation of V_d1ij_ did. The log–log transformation also strongly reduced the spread of the points in the original plot (Poorter and Sack, 2012).

Second, we included all reasonably intact root branches in the model, except root stumps. Specifically, roots branches were considered intact when the sum of distal CSAs was small compared to the proximal CSA (ΣCSA_end_ < CSA/8). To ensure that long, gradually tapering roots were included, we did not remove roots for which V_d_ > CSA × 60 cm. Below 1 cm CSD, the relationship between CSD and √V_d0_ was slightly curvilinear, because below about 2 mm CSD, roots keep approximately the same diameter over several meters (Danjon et al., 2009a). To account for the properties of thin segments, a quadratic term was added to the model:

$$\sqrt{V_{{d0}_{ij}}}=\lambda_{i}+\gamma_{i}{CSD}_{ij}+\beta_{i}{CSD}_{ij}^{2}+\varepsilon_{ij}\,\;\;({QR}_{0})$$

where β_i_ denotes the root type-dependent quadratic effect of the CSD.

Although the residuals of preliminary models were Gaussian-distributed, the variances were still heterogeneous. Consequently, we used a variance power function to account for heteroscedasticity in the models, i.e.,$Var(\varepsilon_{ij})=\sigma^{2}{CSD}_{ij}^{{2\theta }_{i}}$ (Pinheiro and Bates, 2000, p. 211). Note that the parameter θ_i_ in the variance function is root type dependent.

Both models were fitted using a generalized least squares (GLS) estimator (function *gls* in the R package *nlme*; R Core Team, 2012). The two models were compared to simpler nested models using Akaike’s information criterion (Pinheiro and Bates, 2000, p. 84) to ensure that they were not overparameterized (**Table 2**). A potential random effect of tree was also tested using a mixed model approach, but the effect was not significant. The empirical correlations calculated from the within-subject residuals (cf. Fortin et al., 2008) were small in all three datasets, demonstrating that there was no need for a random effect of tree in the models.

**Table 2:** Summary of GLS regression models assessed for descendant root volume.

Dataset	λ_i_	γ_*i*_	β_*i*_	Degrees of freedom	AIC	Log likelihood ratio	Grouping	Model retained
**Structural roots only**								
L5	Global	Global	/	4	13275	13300	a	
L5	Root type	Root type	/	28	12215	12386	b	LR_1_
L5	Root type	Root type	Root type	37	11978	12204	c	
L12	Global	Global	/	4	11525	11548	a	
L12	Root type	Root type	/	31	10390	10571	b	LR_1_
L12	Root type	Root type	Root type	41	10327	10566	c	
L19	Global	Global	/	4	21190	21215	a	
L19	Root type	Root type	/	31	19526	19723	b	LR_1_
L19	Root type	Root type	Root type	41	19493	19753	c	
**All roots**								
L5	Global	Global	/	4	14125	14150	a	
L5	Root type	Root type	/	25	11614	11768	b	
L5	Root type	Root type	Root type	33	11346	11550	c	QR_0_
L12	Global	Global	/	4	12354	12378	a	
L12	Root type	Root type	/	28	8785	8952	b	
L12	Root type	Root type	Root type	37	8663	8884	c	QR_0_
L19	Global	Global	/	4	28561	28588	a	
L19	Root type	Root type	/	28	23973	24159	b	
L19	Root type	Root type	Root type	37	23665	23911	c	QR_0_

*Data were either restricted to root segments from structural roots or included all root segments. ‘Global’ denotes that the parameter was fixed for all root types, ‘Root type’ denotes that it varied by root type, and ‘/’ denotes that it was not included in the model. AIC is Akaike’s information criterion; significant differences between models at a 5% level are indicated as letters.*

The amount of lost roots was then estimated for all broken root ends using the QR_0_ model, because it could be used also for the small CSDs. Because the back transformation of the predictions to the original scale is subject to a bias (Gregoire et al., 2008), we used a naive correction that consisted of adding the prediction error variance to the squared estimate (cf. Fortin et al., 2008).

## RESULTS

**FIGURE 3:**
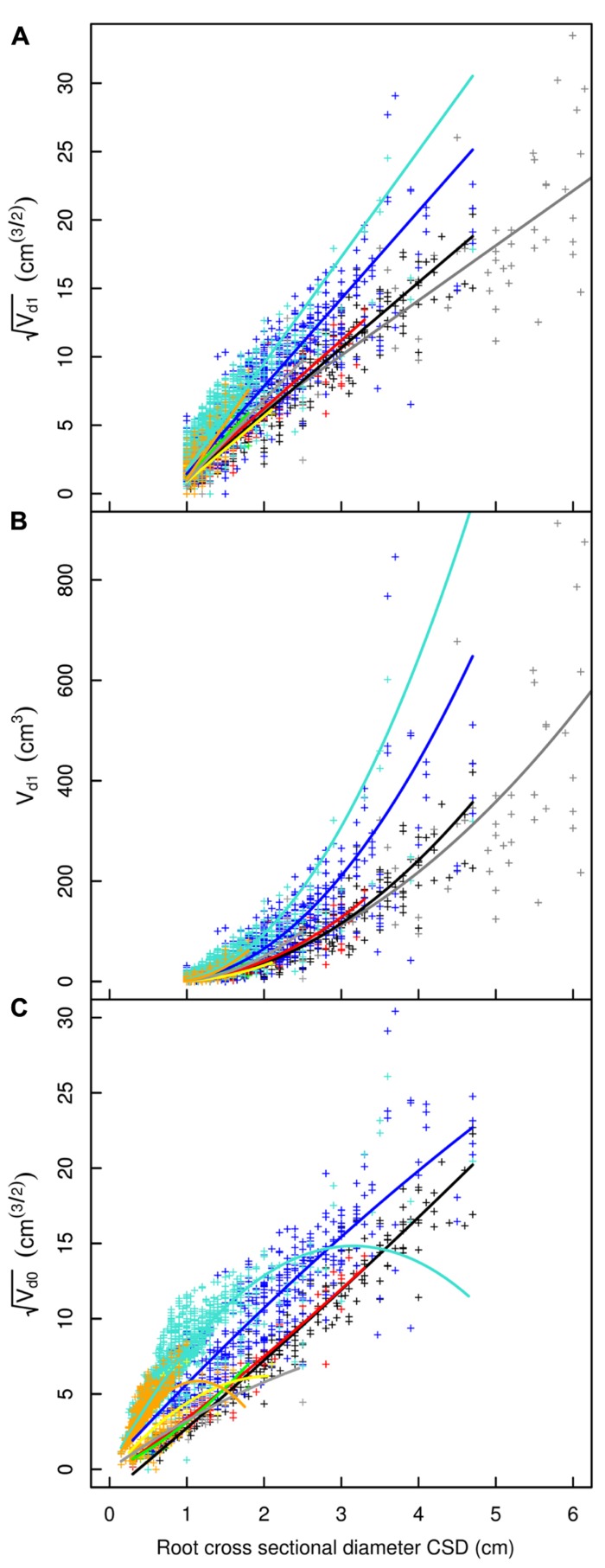
**Descendant volume and cross sectional diameter of root segments in the L5 dataset**. **(A)** LR_1_ regression: intercept and slope vary as a function of root type, applied only to root segments with CSD > 1 cm. **(B)** LR_1_ regression without transformation. **(C)** QR_0_ regression: includes a quadratic term that also varies by root type; applied to all root segments. Coloration follows **Figure 1**, but with higher order shallow roots in orange. The x-axis extends to 6 cm to show the primary area of interest; the cropped black line in **(A)** and **(B)** corresponds to the root stump.

**FIGURE 4:**
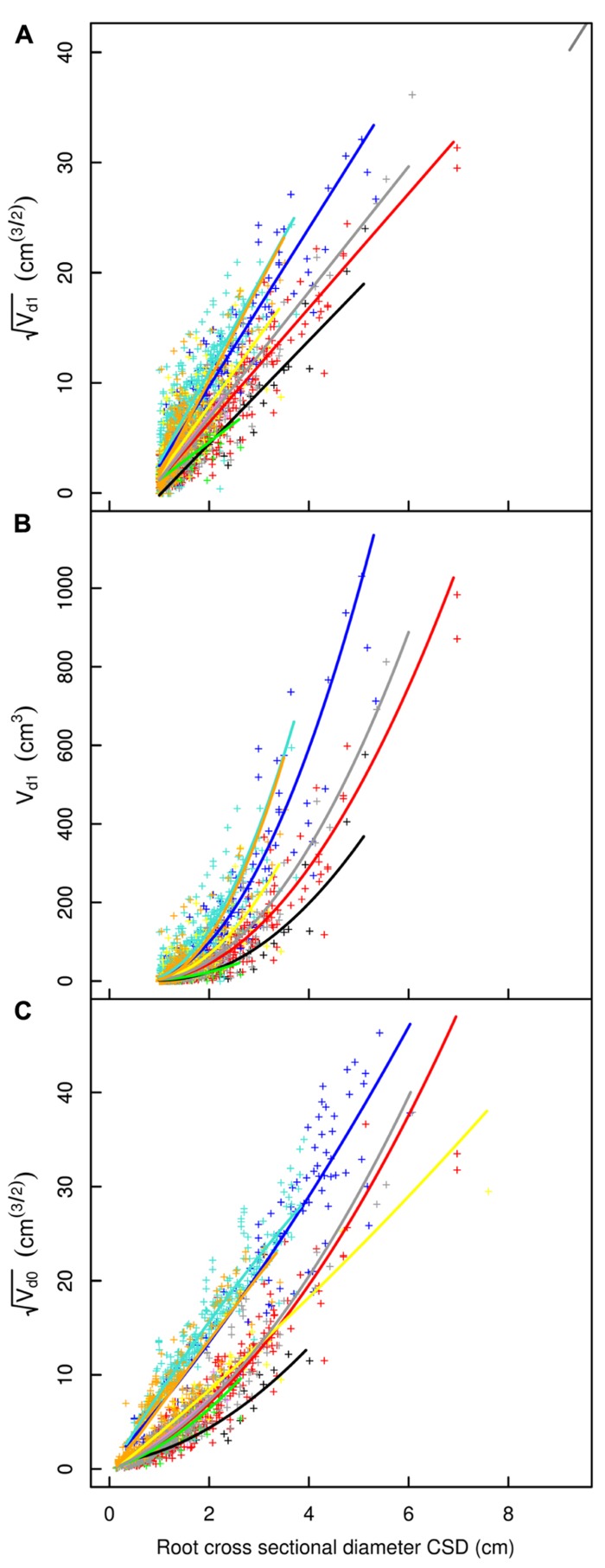
**Descendant volume and cross sectional diameter of root segments in the L12 dataset**. **(A)** LR_1_ regression, **(B)** LR_1_ regression without transformation, **(C)** QR_0_ regression. Coloration follows **Figure 3**. The x-axis extends to 9 cm to show the primary area of interest; the cropped black line in **(A)** corresponds to the root stump.

**FIGURE 5:**
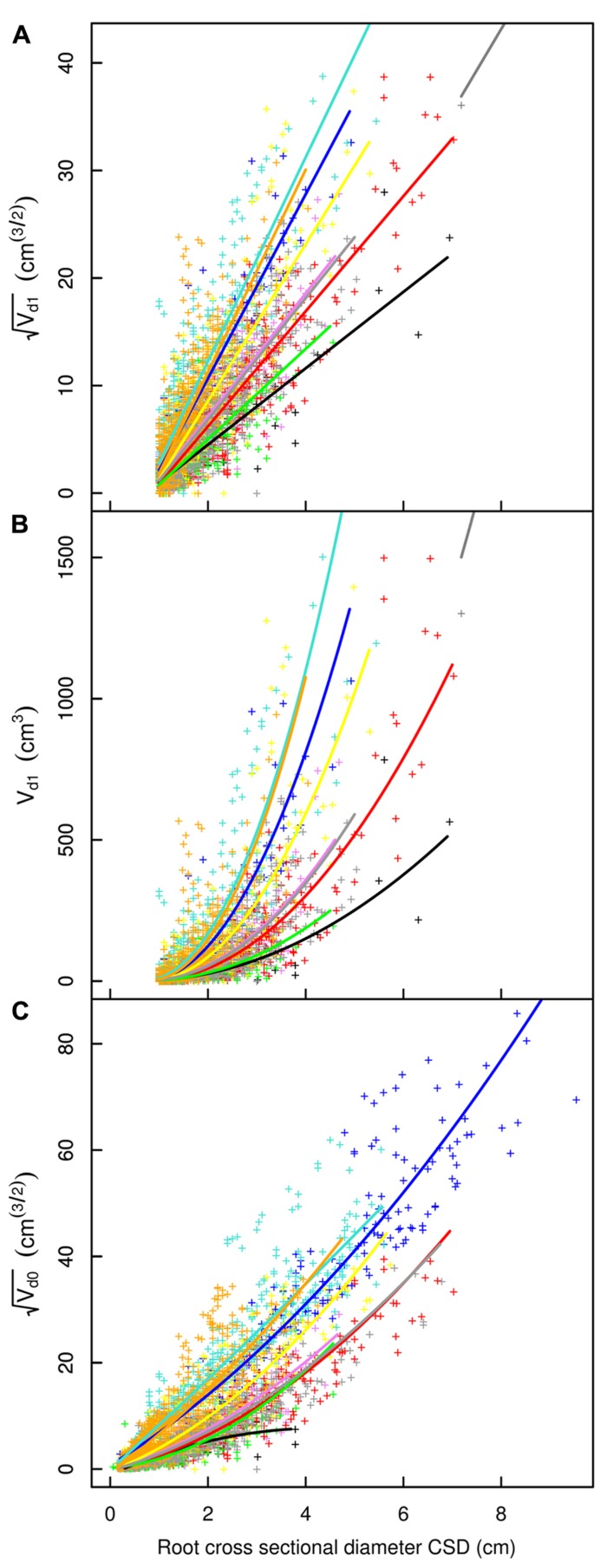
**Descendant volume and cross sectional diameter of root segments in the L19 dataset**. **(A)** LR_1_ regression, **(B)** LR_1_ regression without transformation, **(C)** QR_0_ regression. Coloration follows **Figure 3**. The x-axis extends to 9 cm to show the primary area of interest; the cropped black line in **(A)** and **(B)** corresponds to the root stump.

**FIGURE 6:**
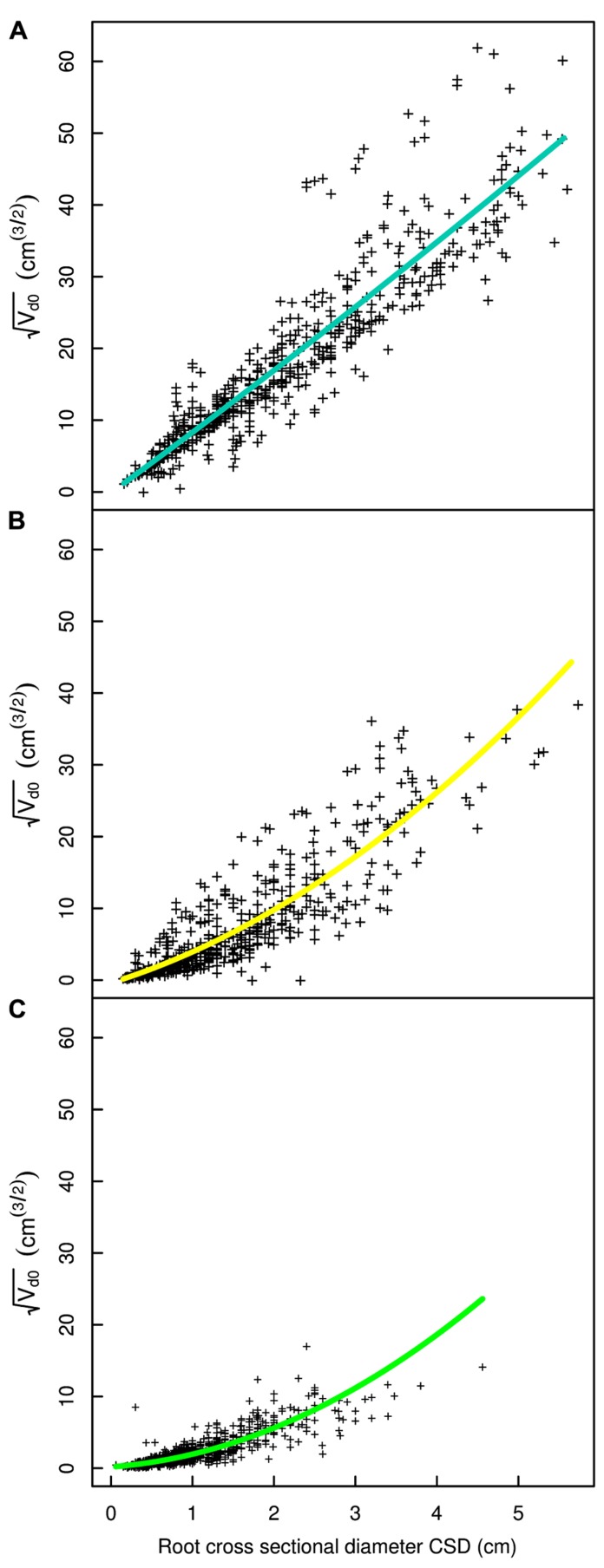
**Regression curves for select root types in the L19 dataset**. **(A)** Shallow second order roots beyond the ZRT, **(B)** intermediate depth horizontals roots, **(C)** deep roots. The QR_0_ regression model is used in all panels. Coloration follows **Figure 3**.

**FIGURE 7:**
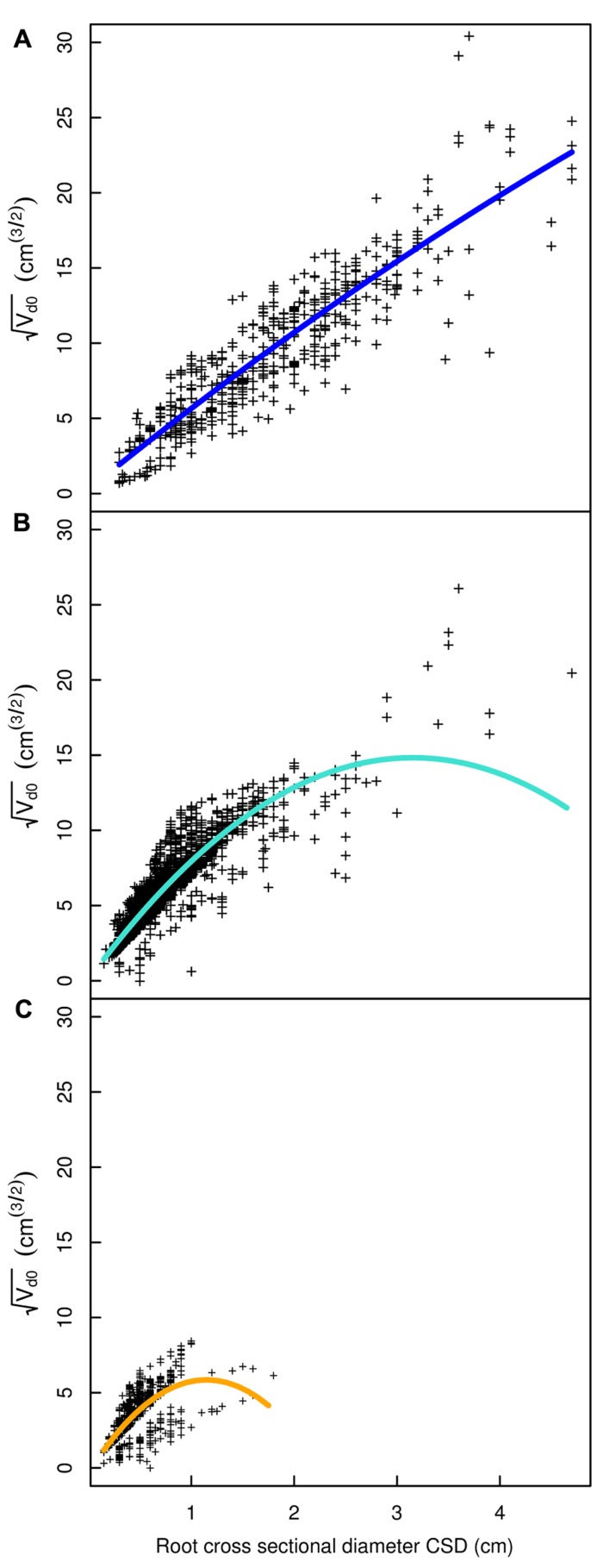
**Regression curves for select root types in the L5 dataset**. **(A)** Shallow roots in the ZRT, **(B)** shallow roots beyond the ZRT, **(C)** higher order shallow roots. Coloration follows **Figure 3**.

A good fit was obtained for all models, except with the QR_0_ model in the L5 dataset (**Figures 3–6; Tables 2–5**). Two curves were parabolic (**Figure 7**): that for shallow roots beyond the ZRT and that for higher-order shallow roots. In these cases, the few roots with the largest CSD had a low descendent root volume. Thus, QR_0_ may not deliver accurate estimation outside the range of CSD values used for estimation. However, this did not cause inaccuracies in estimates of lost volume, because the CSDs of the largest broken root ends were much smaller than the largest CSDs used to develop the models (1.5 cm in shallow roots beyond the ZRT and 1 cm in higher-order shallow roots). As a result of the selection procedure, large root sections within the ZRT itself were not included in LR_1_ (**Figures 3–5**).

**Table 3:** Results of the LR_1_ regression, generalized least squares linear regression per root type between the cross sectional diameter and the square root of descendant root volume for each of the three separate datasets, structural roots only, including the root type-dependent variance parameter. Parameter values and 95% confidence intervals (CI).

**Parameter**	**Root type**	**L5**		**L12**		**L19**	
		**Value**	**CI**	**Value**	**CI**	**Value**	**CI**
λ^i (root type-dependent intercept)	Root stump	-1.92	±1.84	-25.84	±11.58	-18.06	±27.38
	Taproot	-3.74	±0.38	-4.88	±1.61	-2.60	±1.86
	Shallow in ZRT	-4.96	±0.29	-4.69	±0.78	-6.45	±1.50
	Shallow beyond ZRT	-6.03	±0.32	-5.43	±0.67	-6.70	±0.86
	Higher-order shallow	-7.08	±1.48	-7.03	±1.03	-7.60	±0.82
	Sinkers from ZRT	-3.77	±0.49	-4.02	±0.40	-4.49	±0.49
	Sinkers from beyond ZRT	NA	NA	-4.55	±0.80	-4.38	±0.52
	Intermediate depth	-3.90	±0.91	-4.67	±0.79	-6.03	±0.69
	Deep	-4.72	±2.94	-2.23	±1.11	-3.40	±0.54
	Oblique	-4.91	±1.15	-4.51	±0.47	-4.47	±0.40
γ^i (root type-dependent slope)	Root stump	4.01	±0.39	7.15	±0.62	7.65	±1.28
	Taproot	4.80	±0.18	4.68	±0.70	3.55	±0.84
	Shallow in ZRT	6.40	±0.19	7.18	±0.45	8.56	±0.91
	Shallow beyond ZRT	7.78	±0.26	8.21	±0.49	9.48	±0.65
	Higher-order shallow	8.15	±1.32	8.63	±0.81	9.42	±0.64
	Sinkers from ZRT	4.99	±0.32	5.20	±0.24	5.35	±0.26
	Sinkers from beyond ZRT	NA	NA	5.80	±0.55	5.74	±0.34
	Intermediate depth	4.78	±0.63	6.29	±0.59	7.29	±0.49
	Deep	5.90	±2.31	3.43	±0.75	4.20	±0.37
	Oblique	5.87	±0.92	5.69	±0.32	5.65	±0.25
σ^2		1.09	±0.04	1.34	±0.06	1.60	±0.06
θ^i (root type-dependent variance parameter)	Root stump	0.99	±0.07	0.79	±0.07	1.02	±0.09
	Taproot	0.35	±0.10	0.45	±0.29	0.65	±0.24
	Shallow in ZRT	0.85	±0.08	0.73	±0.15	0.98	±0.23
	Shallow beyond ZRT	0.93	±0.13	1.17	±0.17	1.40	±0.14
	Higher-order shallow	1.06	±0.58	1.15	±0.27	1.52	±0.15
	Sinkers from ZRT	0.31	±0.18	0.48	±0.11	0.66	±0.08
	Sinkers from beyond ZRT	NA	NA	-0.26	±0.29	0.59	±0.11
	Intermediate depth	-1.66	±1.29	0.92	±0.21	1.12	±0.11
	Deep	0.27	±2.40	-0.13	±0.35	0.41	±0.13
	Oblique	0.78	±0.35	0.47	±0.13	0.70	±0.08

**Table 4:** Same as Table 3, but with only four root categories: (1) tap root and deep roots, (2) sinkers and oblique roots, (3) shallow roots in ZRT, intermediate depth horizontal roots and root stump, and (4) shallow roots beyond ZRT and higher-order shallow roots.

**Parameter**	**Root type**	**L5**		**L12**		**L19**	
		**Value**	**SE**	**Value**	**SE**	**Value**	**SE**
λ^i (root type-dependent intercept)	Taproot and deep roots	-3.70	0.36	-3.39	0.76	-3.16	0.48
	Sinker and oblique roots	-4.05	0.45	-4.13	0.28	-4.29	0.26
	ZRT, intermediate depth and root stump	-4.37	0.26	-4.47	0.31	-6.09	0.51
	Shallow beyond and higher order	-6.29	0.32	-6.36	0.65	-7.65	0.67
γ^i (root type-dependent slope)	Taproot and deep roots	4.78	0.17	4.19	0.46	4.01	0.32
	Sinker and oblique roots	5.18	0.31	5.36	0.18	5.49	0.16
	ZRT, intermediate depth, and stump	5.85	0.18	6.47	0.20	7.54	0.35
	Shallow beyond and higher order	7.96	0.26	8.73	0.50	9.91	0.52
σ^2		1.07	0.04	1.34	0.06	1.61	0.05
θ^i (root type-dependent variance parameter)	Taproot and deep roots	0.37	0.10	0.36	0.20	0.46	0.11
	Sinker and oblique roots	0.48	0.16	0.45	0.09	0.67	0.06
	ZRT, intermediate depth, and stump	1.12	0.06	0.86	0.07	1.05	0.07
	Shallow beyond ZRT and higher order	1.00	0.13	1.29	0.16	1.52	0.12

**Table 5:** Parameters of the QR_0_ generalized least squares polynomial regression for each root type, between the cross sectional diameter and the square root of descendant volume for each of the three datasets, for all roots. Parameter values and 95% confidence intervals (CI).

**Parameter**	**Root type**	**L5**		**L12**		**L19**	
		**Value**	**CI**	**Value**	**CI**	**Value**	**CI**
λ^i (root type-dependent intercept)	Taproot	-1.60	±0.92	0.56	±1.98	-1.04	±1.56
	Shallow in ZRT	0.27	±0.39	0.46	±0.69	0.76	±0.74
	Shallow beyond ZRT	0.06	±0.16	-0.22	±0.57	-0.13	±0.29
	Higher-order shallow	-0.32	±0.42	-0.78	±0.24	-0.82	±0.17
	Sinkers from ZRT	-0.43	±0.34	-0.15	±0.15	-0.19	±0.18
	Sinkers from beyond ZRT	NA	NA	-0.66	±0.66	-0.26	±0.31
	Intermediate depth	-0.38	±0.99	-0.55	±0.20	-0.40	±0.18
	Deep	-0.07	±0.95	-0.10	±0.09	0.19	±0.12
	Oblique	0.01	±0.82	-0.15	±0.08	-0.29	±0.12
γ^i (root type-dependent slope)	Taproot	4.26	±0.85	0.71	±2.47	4.08	±2.67
	Shallow in ZRT	5.55	±0.57	5.88	±1.11	5.63	±0.87
	Shallow beyond ZRT	9.37	±0.38	8.58	±0.85	8.32	±0.55
	Higher-order shallow	10.78	±1.39	7.41	±0.59	6.92	±0.46
	Sinkers from ZRT	3.62	±1.10	2.20	±0.36	2.03	±0.40
	Sinkers from beyond ZRT	NA	NA	3.27	±1.60	2.70	±0.63
	Intermediate depth	6.28	±2.69	4.24	±0.52	3.55	±0.50
	Deep	2.27	±4.25	1.82	±0.43	0.82	±0.40
	Oblique	3.58	±2.35	2.23	±0.30	2.57	±0.35
β^i (root type-dependent quadratic effect of the cross sectional diameter)	Taproot	0.08	±0.17	0.60	±0.61	-0.48	±0.82
	Shallow in ZRT	-0.17	±0.16	0.31	±0.25	0.49	±0.15
	Shallow beyond ZRT	-1.49	±0.20	-0.33	±0.26	0.10	±0.17
	Higher-order shallow	-4.70	±1.07	-0.10	±0.26	0.50	±0.21
	Sinkers from ZRT	0.17	±0.63	0.68	±0.15	0.64	±0.13
	Sinkers from beyond ZRT	NA	NA	0.37	±0.81	0.60	±0.23
	Intermediate depth	-1.50	±1.59	0.11	±0.22	0.77	±0.20
	Deep	0.92	±4.15	0.71	±0.46	0.95	±0.25
	Oblique	-0.35	±1.31	0.73	±0.22	0.55	±0.16
σ^2		1.39	±0.04	1.21	±0.03	1.76	±0.03
θ^i (root type-dependent variance parameter)	Taproot	0.13	±0.11	0.26	±0.40	0.59	±0.50
	Shallow in ZRT	0.57	±0.08	0.93	±0.10	0.94	±0.08
	Shallow beyond ZRT	0.73	±0.06	0.91	±0.12	1.08	±0.06
	Higher-order shallow	0.31	±0.08	0.52	±0.08	0.99	±0.05
	Sinkers from ZRT	1.46	±0.26	1.05	±0.11	0.91	±0.10
	Sinkers from beyond ZRT	NA	NA	1.13	±0.53	0.72	±0.10
	Intermediate depth	0.23	±0.26	0.89	±0.14	1.07	±0.07
	Deep	1.87	±0.34	1.84	±0.12	1.07	±0.06
	Oblique	0.50	±0.36	1.51	±0.09	1.12	±0.09

Root type strongly influenced the intercepts, first-order, and second-order model coefficients for both models in all three of the datasets (*P* < 0.001; **Figures 3**–**5**; **Tables 3** and **5**). γ_i_ varied from 3.5 to 9 between deep roots and shallow roots. As a consequence, the predicted V_d_ for a 2 cm CSD root in L19 reached 43 cm^3^ in deep roots, 102 cm^3^ in intermediate depth roots, and 290 cm^3^ in shallow, second-order roots beyond the ZRT. Rankings of root categories were fairly consistent across the three datasets. Roots could be roughly divided into four categories according to γ_i_ in LR_1_ (**Tables 3 and 5**) and the shape of the curve in QR_0_ (**Figures 3–5**):

(1) Shallow roots beyond ZRT and higher-order shallow roots, for which γ_i_ seems to increase with age from 8 to 9.5.
(2) Shallow roots in ZRT, intermediate depth roots, and root stump, for which γ_i_ increased approximately with age from 6 to 7.5, except the stump in L5.
(3) Sinkers and oblique roots, for which γ_i_ was near 5.5.
(4) Deep roots and the taproot, for which γ_i_ was near 4, except in L5.

When root types were grouped into four classes (**Table 4**), the above mentioned values for γ_i_ were indeed found, and confidence intervals were smaller.

At the root system level, missing root biomass varied as a function of tree age and of root type (**Table 6**). In younger trees, 12–23% of root biomass was lost per tree, almost exclusively in higher-order roots and in shallow roots beyond the ZRT. The contribution of other root types was marginal. In L12, 2–7% of the root volume was missing at tree level. For L5, most of the missing root volume originated from the shallow roots beyond the ZRT (1.3–3.5%) and in higher-order shallow roots (0–2.3%). In L19, the amount of missing roots varied from 2 to 4% and shallow roots contributed only 70% of the missing volume. In L19, the largest contribution to missing biomass (1066 cm^3^, or 17% of lost volume, in tree no. 5329) came from a single, 3 cm CSD shallow root that was beyond the ZRT.

**Table 6:** Estimation of the percentage of root volume lost downstream of the breaking point, as part of the total volume (above) and within each root type (below).

**Stand:**	**L19**							**L19**		**L12**		**L5**	
**Tree number**	**725**	**4601**	**4824**	**4832**	**4864**	**5306**	**5329**	**Mean**	**SD**	**Mean**	**SD**	**Mean**	**SD**
**Contribution of each root type to root loss**													
Order 1	0.00	0.00	0.00	0.00	0.00	0.00	0.02	**0.0**	0.0	**0.0**	0.0	**0.0**	0.1
Shallow in ZRT	0.06	0.00	0.09	0.01	0.06	0.01	0.00	**0.0**	0.0	**0.1**	0.1	**0.1**	0.2
Shallow beyond ZRT	2.58	2.22	0.95	2.01	1.26	0.47	1.05	**1.5**	0.8	**2.3**	0.8	**9.2**	3.2
High order shallow	0.62	0.45	0.54	1.44	1.42	1.28	0.77	**0.9**	0.4	**1.2**	0.7	**6.2**	2.1
Sinkers from ZRT	0.01	0.03	0.04	0.17	0.02	0.00	0.00	**0.0**	0.1	**0.1**	0.1	**0.1**	0.1
Sinkers from beyond ZRT	0.07	0.19	0.16	0.36	0.30	0.10	0.14	**0.2**	0.1	**0.2**	0.2	**NA**	NA
Intermediate depth	0.09	0.14	0.17	0.22	0.57	0.44	0.31	**0.3**	0.2	**0.3**	0.2	**0.0**	0.1
Deep	0.03	0.19	0.08	0.11	0.08	0.10	0.04	**0.1**	0.1	**0.1**	0.1	**0.8**	0.8
Oblique	0.16	0.29	0.11	0.19	0.17	0.15	0.26	**0.2**	0.1	**0.3**	0.2	**1.0**	1.9
**Total tree**	**3.61**	**3.51**	**2.15**	**4.53**	**3.86**	**2.56**	**2.60**	**3.3**	0.8	**4.4**	1.5	**17.5**	5.1****
***LR0WC % difference***	***-24%***	***-14%***	***+1%***	***-17%***	***-11***	***+6%***	***-13%***	***-10%***	*11*****	***-46%***	*7.8*****	***+86%***	*23*****
***QR0WC % difference***	***-44%***	***-28%***	***-13%***	***-29%***	***-27***	***-12%***	***-14%***	***-23%***	*11*****	***-47%***	*8.7*****	***+64%***	*19*
**% loss in each class**													
Order 1	0.01	0.00	0.00	0.01	0.00	0.00	0.41	**0.1**	0.2	**0.0**	0.0	**0.4**	0.7
Shallow in ZRT	0.26	0.00	0.75	0.07	0.28	0.04	0.01	**0.2**	0.3	**0.2**	0.2	**1.7**	7.0
Shallow beyond ZRT	14.50	11.70	6.45	14.30	10.90	5.21	9.75	**10.4**	3.6	**15.1**	4.2	**27.2**	9.8
High order shallow	13.50	11.30	13.60	9.76	18.30	13.40	5.52	**12.2**	4.0	**22.0**	11.4	**56.1**	9.4
Sinkers from ZRT	0.10	0.54	0.38	1.30	0.17	0.04	0.04	**0.4**	0.5	**0.8**	0.6	**11.5**	13.6
Sinkers from beyond ZRT	5.55	3.64	16.20	9.56	8.98	4.18	2.95	**7.3**	4.7	**11.5**	7.6	**NA**	NA
Intermediate depth	2.87	10.40	3.71	4.57	13.20	8.64	3.57	**6.7**	4.0	**7.7**	5.0	**34.2**	22.3
Deep	3.41	3.69	13.60	4.03	2.86	4.90	6.96	**5.6**	3.8	**9.3**	9.5	**51.0**	22.5
Oblique	4.68	4.05	4.67	2.91	4.47	1.86	3.42	**3.7**	1.1	**8.6**	7.5	**40.7**	18.5

*LR0WC and QR0WC: % difference in estimation of total root loss without taking into account root types compared to estimations obtained using QR_0_. Left: estimation for each tree in L19. Right: mean and standard deviation in L5, L12, and L19. Bold values are summary values. Italicized values correspond to analyses which do not take root types into account.*

The percentage of missing root volume in each root type varied widely (**Table 6**). It was generally close to zero for the taproot, shallow roots within the ZRT, and sinkers below ZRT; these three root types form the central part of the root system. Missing volume had high mean values for shallow second-order roots beyond the ZRT, decreasing with age from 27 to 15 to 10%, though it was still larger in higher-order shallow roots. The amount of roots lost in the other categories varied between the values for the two aforementioned categories, namely around 8% in L12 and L19, with a high inter-tree variability. L5 trees possessed only a few roots in the intermediate depth, deep, and oblique classes, but these were largely broken. When root types were not used for estimation, the percentage of missing root volume was largely underestimated in L12 and L19, but largely overestimated in L5 (**Table 6**).

## DISCUSSION

This study has shown that the relationship between root CSD and descendent volume varies largely as a function of root type. In quantifying these relationships, we have demonstrated that it is possible to accurately estimate the amount of missing root material from datasets describing 3D root system architecture. Estimation is possible provided that the root dataset contains sufficient information on reasonably intact root axes and segments from which to derive a relationship. Basing the estimate on volume makes for a significant time savings over methods that require biomass to be measured for roots of varying sizes. Root loss estimations are much more accurate when roots are classified by type, and the numerical relationships used to estimate missing volume are stratified according to these classes. Root volume data can be subsequently converted to root biomass if necessary, provided that root wood density data are available (Danjon et al., 2008). Given that root tissue density varies little by root type, if at all (it did not vary significantly for mature *P. pinaster* (Danjon et al., 2006), the effort needed to collect appropriate root density measurements will generally be minimal.

Parameter estimates for the model with structural roots only (LR_1_) were highly reliable because only complete branches were used, excluding the root stump. In contrast, for QR_0_ estimates, all of the branches used were broken. Given that quadratic models are flexible, they should not be used outside the range of the data used to estimate their parameters. A quadratic term was needed to cope with the curvature of the relationship for small values of CSD. Defining the optimum criteria to distinguish weakly vs. strongly broken branches is not without challenges. Roots within the ZRT that had large cross sections were not included in the LR_1_ model because at least one root with a broken tip >1 cm generally descended from them.

### VARIATION IN TAPER

The largest difference in γ_i_ was between deep roots and shallow roots beyond the ZRT. For a given diameter in the L19 dataset, LR_1_ estimates that deep roots had about six times less (and sinkers had about two times less) root volume descending from them than shallow second-order roots beyond the ZRT. In FBA studies, the taper at branches, when mentioned (Soethe et al., 2007), is close to 1 (i.e., the sum of CSAs proximal and distal to the branch are equal). This is in accordance with the pipe model (Shinozaki et al., 1964). Thus, the slope γ_i_ mainly accounts for tapering between branches of all descendant root segments in an integrative way and also to branching in fine roots. This means that deep roots, the taproot, and to a lesser extent sinkers and oblique roots, have a high overall tapering rate. This probably occurs for two reasons: (1) shallow roots develop more rapidly because they have a significantly higher potential to contribute to plant productivity than deep roots (Korndoerfer et al., 2008), and (2) root growth is restricted by unfavorable soil conditions in deeper soil horizons (e.g., low nutrient content, hard pans, or water tables). It is therefore surprising that CSA has been considered a good predictor of descendant root biomass in the literature. One reason is that this relationship was mainly assessed in a single root type, with the proximal diameter used for estimation located in the ZRT. The other reason is that a high correlation can simply be caused by a wide range of CSA size values (Poorter and Sack, 2012). For example, in Drexhage and Colin (2001), where the root CSA ranges from 2 to 120 cm^2^, the *r*^2^ is around 0.9 but there is still large variation that is orthogonal to root CSA. Kalliokoski et al. (2008) and Heth and Donald (1978) also found a very large variation orthogonal to root CSA (close to 1:10). It should also be noted that, if γ_i_ varies as a function of root type, FBA parameters will also vary as a function of root type (Kalliokoski et al., 2010). In *R. pseudoacacia* seedlings, tapering between branches scored around 7% for the root stump, 20% for the taproot, and only 5% in laterals (Khuder, 2007).

Variability in taper among root types may be larger than what we computed. For example, large horizontal shallow roots with gradual taper can branch into secondary sinkers with moderate taper, which themselves can branch into deep roots with steep taper. Moreover, even if the branches kept for QR_0_ computations were less broken than the others, they were still broken. Therefore, γ_i_ for unbroken shallow roots without sinkers is probably distinctly larger than 9. In the same way, the descendent volume of ZRT segments also included a large amount of shallow roots beyond the ZRT. That is why the differences in γ_i_ between the two root types were small even though taper in the ZRT was steep. Because deep roots did not bear other types of roots, their γ_i_ was close to 3.5 in limiting soils. Deep roots that tapered steeply were observed in the field, especially in the vicinity of the hard pan.

The large variability of V_d_ for first-order roots can be explained by the structure of the root. For mechanical reasons, the top of the first-order root (the root collar) has a diameter that is generally larger than the DBH of the tree (Coutts, 1987). Below-ground, the diameter increases in the zone where large shallow roots originate (the root stump), with a large diameter maintained through the region where shallow roots originate. The first-order root tapers strongly below this point to form the taproot. Prediction of V_d_ in the first-order root would therefore be unreliable using a single set of parameters for both the root stump and the taproot. In our database the root stump was never broken, so there was no practical reason to estimate its descendent volume.

All trees in this study were from the same species, the same genetic provenance, and grown in the same stand. As no tree effect was detected, these trees probably exhibit low intra-population genetic variability, low plasticity to the micro-environment, and a minimal effect of tree size for the studied relationship. A small but significant increase in γ_i_ was observed with age for certain root types. However, we could not assess whether there is a global age effect, because, in L5, the number of segments was only sufficient to provide reliable estimates in the first-order root and in shallow roots.

A further improvement of our method would be to split the estimates of lost root volume into root types or root diameter categories (Le Goff and Ottorini, 2001). This technique could also be used to estimate structural root length and root number. However, fine roots make up a large proportion of root length and number. It could also be used to estimate the number of fine roots branching from the structural roots, possibly on a sub-sample of intact root branches carefully excavated and digitized, including counts of fine roots. A recurrent procedure could be used to compute parameters of QR_0_ from all the segments of the root systems whether they carry large broken root ends or not. This would entail attributing a lost root volume (from QR_0_) to all broken root ends of the database, and recomputing a corrected descendant root volume for each segment.

### ROOT LOSS IN LARGER TREES

The low fraction of missing roots in our database can be partly attributed to the fact that we worked in shallow, sandy soils, excavated during the wet season, and dealt with root systems that were mainly composed of long, shallow roots and sinkers. The amount of lost roots is expected to be larger without preliminary removal of understory plants and soil preparation. Root loss would also be large in stony, hard, or deeper soils, or for heart root systems (i.e., those with a large proportion of oblique roots originating from the root stump; Danjon et al., 2013). Even in deep soils with deep-rooting species, only few structural roots can be found below a 4 m depth (Christina et al., 2011). While low, the root biomass losses reported here may be slight overestimates. If our assumption of constant root tissue density was incorrect, and density decreased with distance from the root collar (Danjon et al., 2006), biomass in distal roots would have been lower than reported.

The reason that most of the structural root biomass could be easily extracted is that a rigid structure is formed in *P. pinaster* by the root stump, taproot, shallow roots within the ZRT, and sinkers originating from the ZRT; these classes also made up the largest portion of the root biomass (Danjon et al., 2005). When root systems were extracted vertically, the taproot usually remained intact and the large sinker roots originating within the ZRT were also recovered. However, much larger losses occurred in the shallow roots beyond the ZRT. After we removed understory vegetation, litter, and the upper soil surface, most of the thicker shallow roots were removed within a radius of 3 m by manual pulling. However, as these roots taper very gradually, about 15% of their volume was still lost in larger trees. As a point of comparison, in a prior study that used four *Fagus sylvatica* trees (Le Goff and Ottorini, 2001), the estimate of lost root biomass varied from 5 to 35%. Heth and Donald (1978) experienced an average loss of 1.6% in 38 cm DBH *Pinus radiata* trees and an average loss of 10.6% in 47 cm DBH trees. Niiyama et al. (2010) reported a 23% mean loss in a sample of 121 tropical trees ranging from 0.4 to 116 cm DBH, and suggested that the proportion of root loss increases with tree size. We observed the opposite relationship with tree size. Our uprooting technique was more efficient for larger trees, and structural roots of the L5 trees broke more easily than those of L12 and L19.

### APPLICATION

The approach described here to estimate root losses during uprooting can be used to improve estimates of forest carbon storage. Regional carbon stocks in forests are quantified using stem volume measurements that are taken during forest inventories. Carbon stored in root systems are incorporated through a biomass expansion factor (FEB), or the ratio between total biomass and stem biomass. An estimate of FEB in a mature *P. pinaster* stand that did not account for missing roots yielded a value of 1.585 (Bert and Danjon, 2006). However, using the 4% coarse root loss found here, the corrected FEB would increase to 1.598. Similarly, the ratio between total woody biomass and aboveground woody biomass, referred to as the root expansion factor (REF) originally scored 1.26, but would rise to 1.27 after being corrected. The original root mass fraction (RMF) of 20.6% would change to 21.2% after correction. Correction for missing roots is also useful for assessing the percentage of root biomass or mineral mass exported from a forest by root system harvesting (Augusto et al., 2013).

Root loss diminishes strongly with increasing diameter. Therefore, during uprooting of larger trees, one must avoid losing roots with CSD larger than about 1.5 cm, especially in shallow roots with large γ_i_. A better estimation of γ_i_ and lost root biomass in shallow roots may have been achieved by carefully excavating a few shallow roots per tree before uprooting.

In some cases, model coefficients may need to be derived from a different set of plants than those for which missing volume is estimated. This is the case in plants that are excavated quickly and in which a large amount of root material is lost. For example, rapid uprooting is necessary in studies using high throughput phenotyping (Danjon et al., 2009b; Trachsel et al., 2011). An alternative to using separate plants for missing volume estimation would be to carefully uproot and digitize a small, stratified sample of roots specifically for deriving regression parameters for the root types of interest.

### ROOT SYSTEM DIFFERENTIATION

The fact that roots experience diverse growth conditions, even within a single plant, may limit the applicability of conventional fractal branching models (e.g., Soethe et al., 2007) in problems like estimating the root volume lost in excavation. For instance, root tapering and related fractal properties may be altered by growth responses to mechanical stimuli (Danjon et al., 2005), soil geometry (Nicoll et al., 2006), soil layer properties, and resource availability (Pierce et al., 2013). Models that account for variation in root morphology and architecture, especially those accounting for root types (Jourdan and Rey, 1997; Collet et al., 2006) are probably better suited to reconstructing root biomass from partial measurements of root systems. This study also demonstrates that the prediction accuracy of root properties from proximal diameter measurements is substantially higher when multiple trees and whole root systems are used for analysis. FBA has generally been performed with modest numbers of branching points (250 branching points in Richardson and Dohna, 2003, 200 per species and stand in Kalliokoski et al., 2010). Descendent volume has also been assessed with modest numbers of root samples (27 in Niiyama et al., 2010; 400 in Heth and Donald, 1978; 100 in Le Goff and Ottorini, 2001; three per tree in 417 trees from four species in 20 stands in Nielsen and Hansen, 2006).

High variability in taper between root types is likely to be observed in most woody species, given that roots can generally be classified as shallow, sinker, and deep roots. There was high variability in the database used herein, despite the fact that it described a single, monospecific stand with a single provenance. Exceptions may be found: for example, roots specialized for starch storage, roots with a specialized mechanical function (the taproot in *Q. petraea*; Reubens et al., 2009), or adventitious roots originating in stems, as in the banyan, mangrove, and certain orchids. The datasets of Kalliokoski et al. (2010) and Nielsen and Hansen (2006) suggest that there can also be a large inter-species and inter-soil-type variability for γ_i_ .

Insofar as it describes a rate of tapering, the magnitude of γ_i_ may vary with root type consistently across species. For example, the magnitude may correspond to the strategy used for anchorage, water uptake, or nutrient absorption. These parameters may therefore have similar utility to topological indices, which describe the connectivity of branches along the spectrum from herringbone to dichotomous, and the associated soil exploration and exploitation potentials (Fitter, 1987). The variability of γ_i_, e.g., among species within a genus or among ages within a species, may be useful indicators of the degree to which roots are differentiated into multiple functional classes. One approach would be to assess the degree of differentiation for each root system of interest using the ratio of γ_i_ for a reference type (e.g., distal shallow roots) and γ_i_ of a given root type. By this measure, differentiation for deep roots scored 2.3 in the studied stand. Limitations with respect to inferring and comparing below-ground strategies using this approach include the fact that root taper is difficult to measure and the inconsistency of root types among species. While it appears that the γ_i_ of proximal structural roots is mainly associated with anchorage, and the γ_i_ of shallow distal roots is mainly associated with absorption, the basis of γ_i_ must be better understood before it can be used to infer functional significance and the degree of differentiation among root types. Also, the variability of γ_i_ for each root type can be better characterized. To better understand the variation in the relationship between CSD and V_d_, the variability of fractal branching parameters (mainly taper between branches) in the root system, and its variation as a function of root type should be examined. Further research will be needed, spanning multiple species and wide array of substrate conditions.

## Conflict of Interest Statement

The authors declare that the research was conducted in the absence of any commercial or financial relationships that could be construed as a potential conflict of interest.
